# Pre- and Postoperative MRIs of a Case of Esotropia With an Accessory Extraocular Muscle

**DOI:** 10.7759/cureus.90813

**Published:** 2025-08-23

**Authors:** Akari Arakawa, Miho Sato, Tadahiro Tokushima, Asako Ito, Miwa Komori, Hiroki Kaneko, Akiko Hikoya

**Affiliations:** 1 Department of Ophthalmology, Hamamatsu University School of Medicine, Hamamatsu, JPN; 2 Department of Ophthalmology, Iida Municipal Hospital, Iida, JPN; 3 Department of Rehabilitation, Clinical Technology Division, Iida Municipal Hospital, Iida, JPN

**Keywords:** accessory extraocular muscle, atypical strabismus, congenital abnormalities, mri images, optic nerve, postoperative

## Abstract

Accessory extraocular muscles are rare abnormal structures that attach to the posterior globe, resulting in atypical limitations of eye movement and enophthalmos. We present herein the case of a 12-year-old girl with severe esotropia and restricted abduction in the right eye, who was found to have an accessory extraocular muscle on MRI. The patient underwent a successful surgery, and a comparison of pre- and postoperative MRIs showed improvement in the right eye’s position and release of the abnormal structure in the orbit. To the best of our knowledge, this is the first reported case comparing pre- and postoperative MRI findings of an accessory extraocular muscle.

## Introduction

Accessory extraocular muscles, which are frequently found in animals such as horses, pigs, and sheep, are rare in humans [1,2]. Clinical features associated with accessory extraocular muscles include strabismus with atypical restriction of eye movement and enophthalmos, presenting similarly to congenital cranial dysinnervation disorders (CCDDs), such as Duane retraction syndrome, Möbius syndrome, and Ciancia syndrome, as well as congenital fibrosis of the extraocular muscle. Diagnostic imaging of the orbits is beneficial in obtaining a definitive diagnosis of accessory extraocular muscles [3,4].

Accessory extraocular muscles are often located deep in the orbit close to the optic nerve; therefore, even if a diagnosis is made based on imaging findings, the surgery is often not performed, or patients may not show improvement in eye position after surgery [4,5]. At present, however, there is no literature describing postoperative MRIs of the accessory extraocular muscle. We report herein a case of esotropia (ET) caused by an accessory extraocular muscle in which surgery was successfully performed, and pre- and postoperative MRI images were compared.

## Case presentation

**Figure 1:**
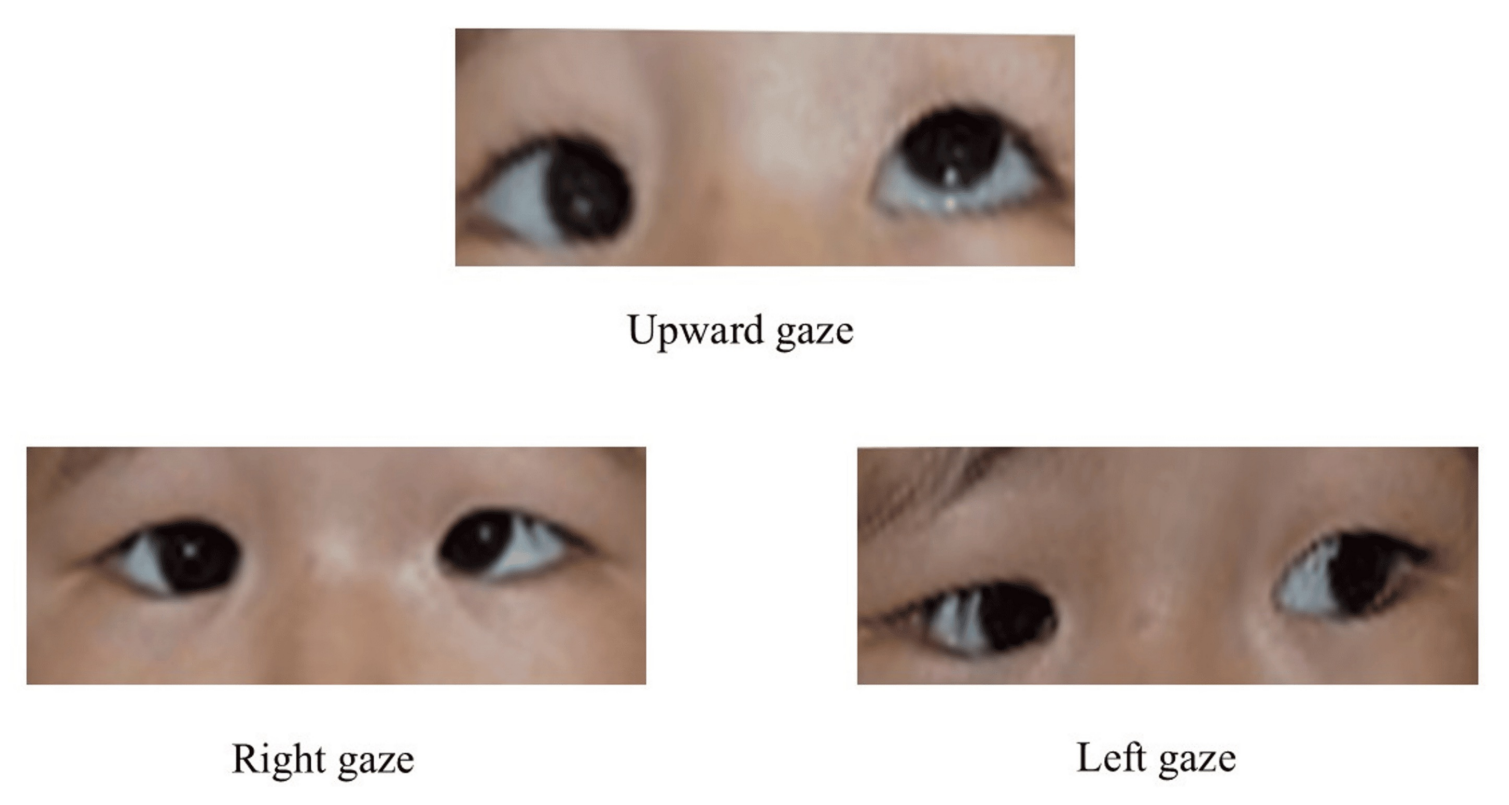
Eye movements at the time of the first visit to our clinic at 14 months of age The right eye is markedly restricted in abduction, as well as in adduction and supraduction. The guardian consented to have the patient's identity revealed in an open-access publication. A signed consent statement was provided to the journal.

A 14-month-old girl was brought to us for an evaluation after her family noticed that her eyes were abnormally positioned when she was approximately six months old. She was diagnosed with ET of the right eye and was referred for further evaluation and treatment. She had no other medical history nor a family history of eye diseases. Her visual acuity was 0.3 (cycles/degree, 38 cm) in the right and 1.1 (cycles/degree, 38 cm) in the left with the Teller Acuity Cards. There were no significant refractive errors, and no clinical findings were observed in the anterior segment or fundus of either eye. The Hirschberg test demonstrated approximately 15° of ET and hypotropia in the right eye. Motility examination demonstrated limited abduction in the right eye (Figure 1).

**Figure 2:**
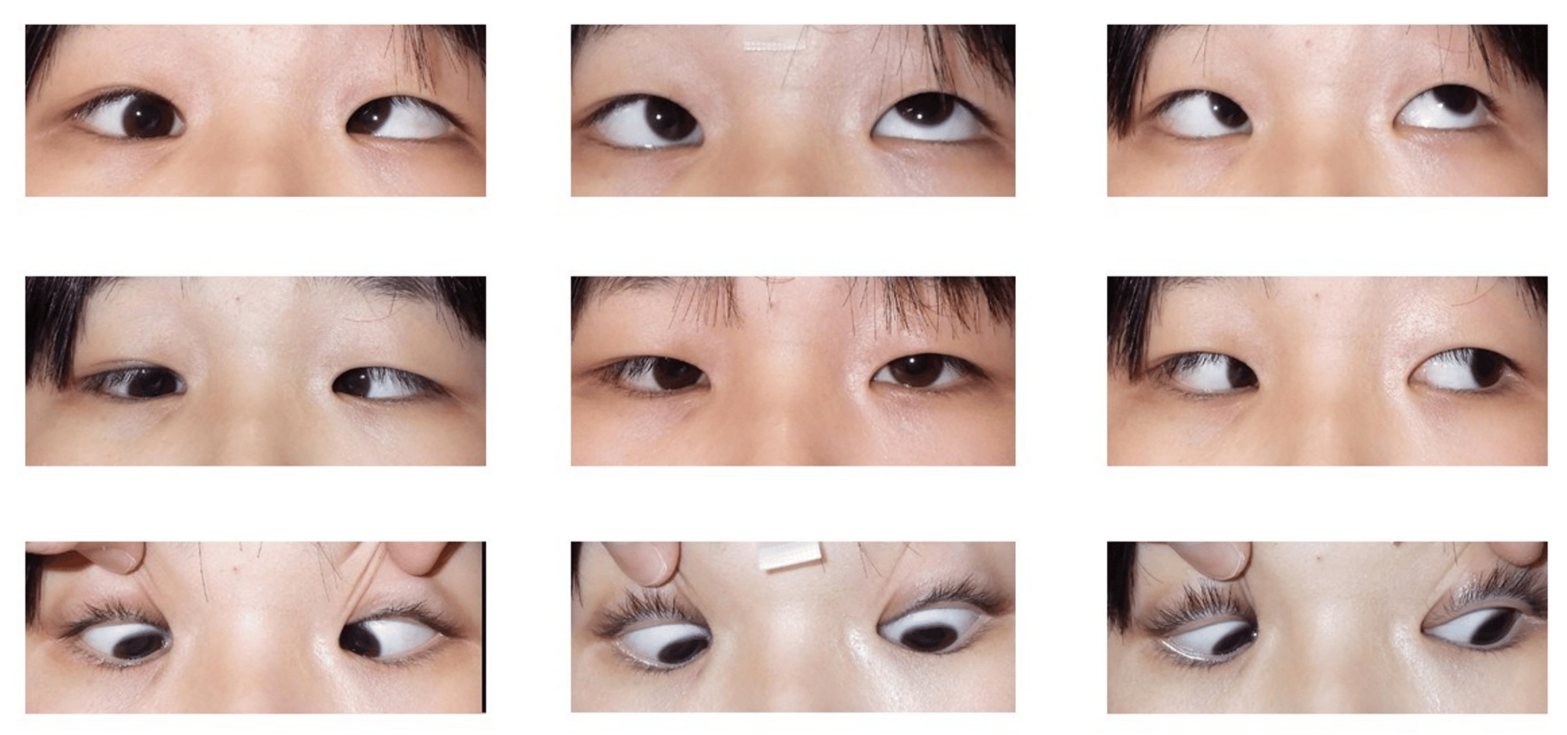
Eye movements at 12 years of age In the right eye, limitations of abduction (-4) and supraduction (-2), as well as downshoot during abduction efforts, were observed. The guardian consented to have the patient's identity revealed in an open-access publication. A signed consent statement was provided to the journal.

There were no obvious intracranial abnormalities on a head MRI; however, an orbital MRI revealed an abnormal structure branching from the right medial rectus muscle (MR) and connecting to the orbit. Treatment of amblyopia of the right eye by patching the left eye was recommended. Because the patient lived far from our hospital, she was followed up by a local ophthalmologist and returned to our clinic at 12 years old for the treatment of her ET. At that time, her visual acuity was 20/400 (not correctable (n.c.)) in the right and 20/15 (n.c.) in the left. The Krimsky test showed 45 prism diopters (PD) ET in the right eye. Intraocular pressure (IOP) measured 17 mmHg in the primary position of the right eye, increasing substantially to 30 mmHg during abduction attempts. The right eye was restricted in abduction (-4) and supraduction (-2), and showed downshoot during abduction attempts (Figure 2).

**Figure 3:**
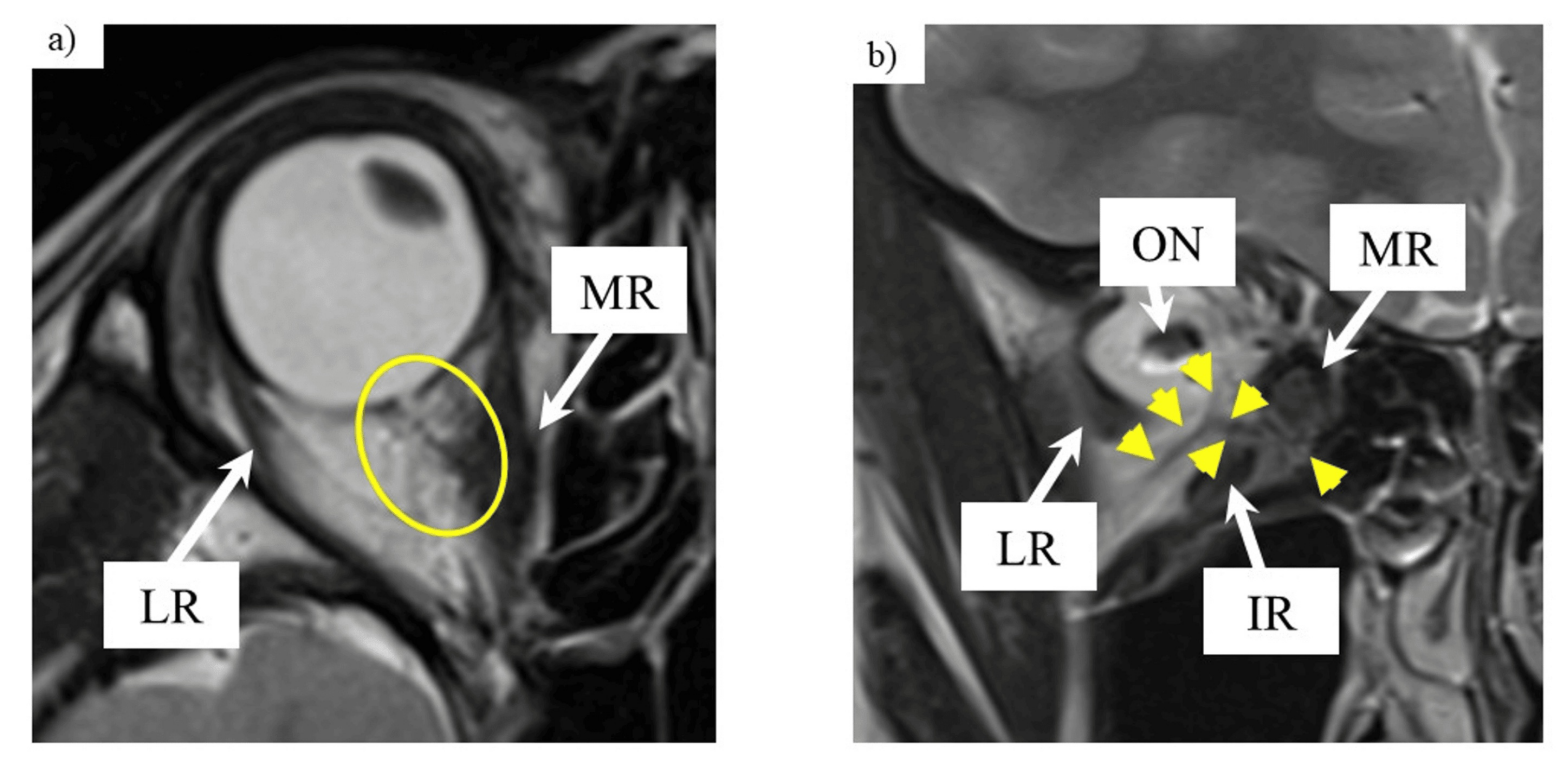
MRI of the right orbit obtained at 12 years of age (a) Axial T2-weighted MRI. An accessory extraocular muscle is seen extending from the right MR to the posterior orbit (yellow circle). (b) Coronal T2-weighted MRI. A diffuse abnormal structure is visible between the MR and IR, with signal intensity intermediate between adipose tissue and extraocular muscle (yellow arrows). This tissue was identified as an accessory extraocular muscle. All images are 3.0-mm thick. LR: lateral rectus muscle, ON: optic nerve, MR: medial rectus muscle, IR: inferior rectus muscle, MRI: magnetic resonance imaging

Another MRI of the orbit was performed, which showed an abnormal structure in the area between the MR and inferior rectus muscle (IR), which was connected to the orbit from the MR (Figure 3). The patient was diagnosed with ET with an accessory extraocular muscle.

Strabismus surgery was performed under general anesthesia. Abduction was significantly restricted in the right eye during the intraoperative traction test. We placed stay sutures, made a limbal incision in the conjunctiva on the nasal side, and opened the sub-Tenon’s space. There was a thin but tight fibrous tissue attached to the globe at the 3 o’clock position, mimicking the MR. Although this structure was dissected from the sclera with scissors, eye movement during attempted abduction did not improve. We subsequently looked deeper and found another fibrous tissue attached to the sclera. It was very deep and could not be hooked; therefore, it was removed from the sclera using scissors while pressing with a cotton swab. After releasing the structure from the globe, the eye was able to be manually rotated beyond the midline. The conjunctiva was sutured, and the surgery was concluded (Video 1).

**Video 1:** Intraoperative traction test (right eye, surgeon’s view) During the intraoperative traction test, the right eye was fixed on the nasal side with limited abduction and supraduction. No limitation was noted in the left eye. A stay suture was placed, and a limbal incision was made on the nasal side. The conjunctiva and Tenon’s capsule were dissected from the sclera, and the muscle presumed to be the MR was identified. The MR was detached from the sclera using scissors, with no improvement in motility. Another fibrous tissue arising from the deep orbit was found after blunt dissection. The second fibrous tissue was detached from the globe using a cotton swab. The right eye was abducted until it passed the midline. MR: medial rectus muscle

**Figure 4:**
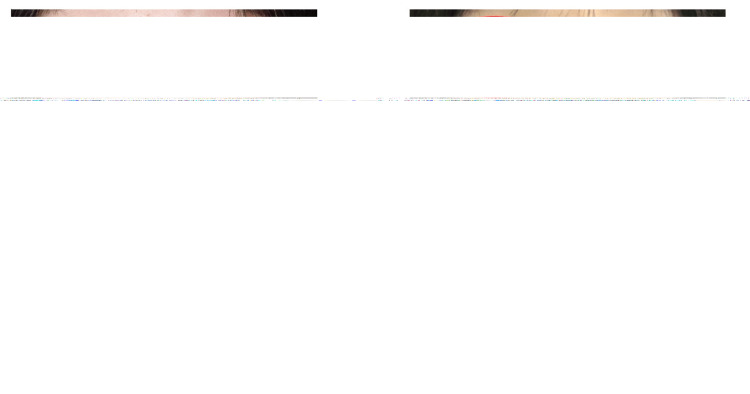
Pre- and postoperative photographs of eye movements Left: preoperative. Right: postoperative. After surgery, the right eye showed improved abduction (-3) and supraduction (-1), and the downshoot during abduction was no longer observed (red circle). The guardian consented to have the patient's identity revealed in an open-access publication. A signed consent statement was provided to the journal.

One week after surgery, the visual acuity was 20/200 (n.c.) in the right and 20/16 (n.c.) in the left. The Krimsky test showed 20 PD ET in the primary position. Right eye movement was restricted in abduction (-3) and supraduction (-1), although downshoot was no longer observed during abduction (Figure 4).

**Figure 5:**
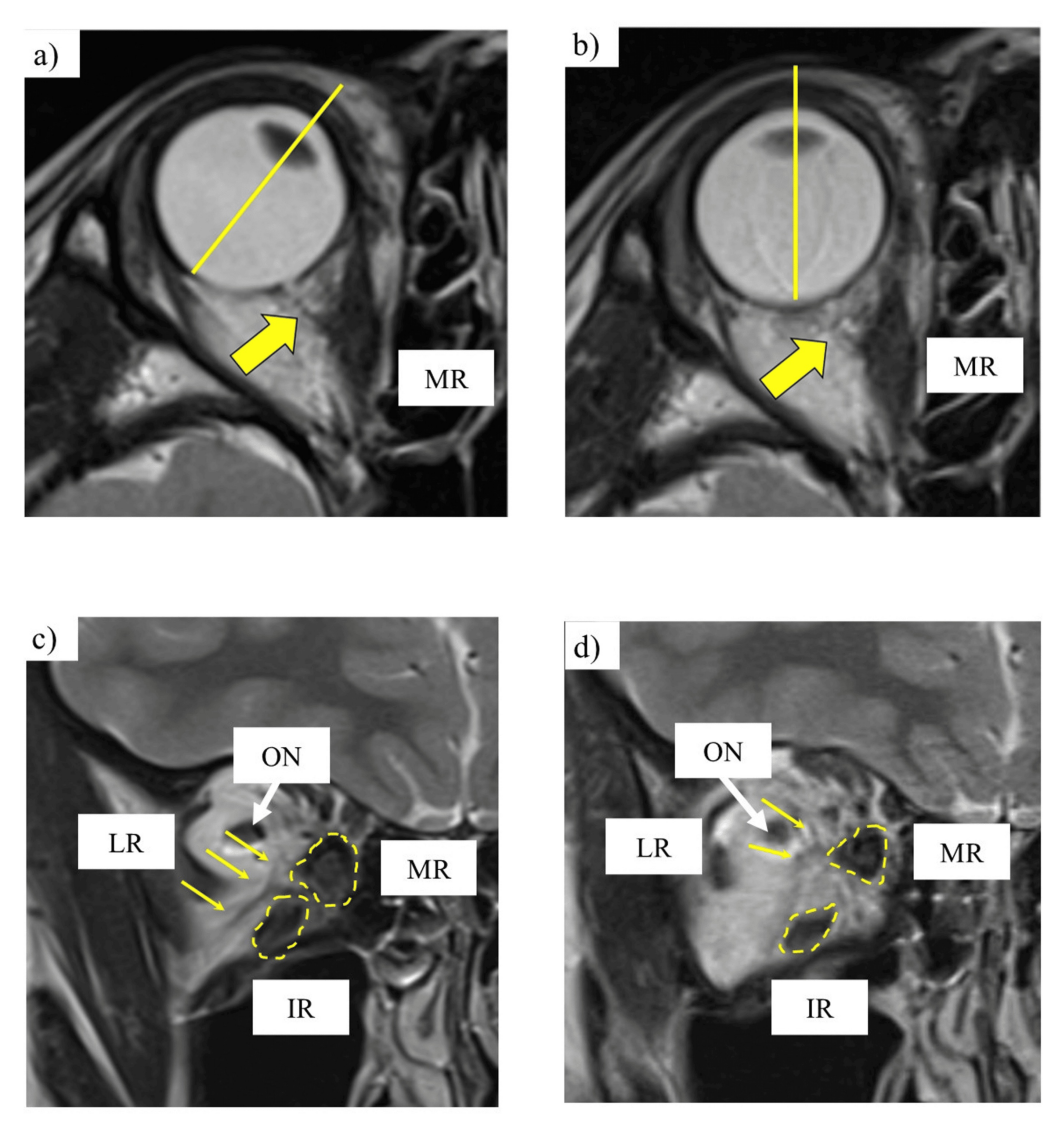
Pre- and postoperative MRIs of the orbit (a, b) Axial T2-weighted MRIs before and after surgery. (c, d) Coronal T2-weighted MRIs before and after surgery. Postoperatively, the accessory extraocular muscle (yellow arrows) previously attached to the posterior aspect of the globe from the MR appeared relaxed (a, b), thinner, and displaced upward. The bellies of the MR and IR (yellow dot circles) also appeared thinner (c, d). All images are 3.0-mm thick. LR: lateral rectus muscle, ON: optic nerve, MR: medial rectus muscle, IR: inferior rectus muscle, MRI: magnetic resonance imaging

The IOP was 12 mmHg in the primary position and 13 mmHg during abduction, with no further elevation of the IOP, indicating that the mechanical restriction was resolved. Postoperative MRI of the orbit revealed the detachment of the accessory extraocular muscle from the globe. The space between the MR and IR was also separated, and each muscle was thinner than before surgery. We confirmed that the right eye was oriented in a straight position (Figure 5).

## Discussion

Characteristic MRI findings of accessory extraocular muscles have been described by Lueder [2], who classified these structures into three types based on imaging findings: (1) structures arising from the extraocular muscles themselves and inserted in abnormal locations, (2) fibrous bands located beneath the rectus muscles, and (3) discrete abnormal structures arising from the posterior orbit. Additionally, Khitri and Demer [3] reported a case series of MRI findings in accessory extraocular muscles, describing them as tissue smaller than but bridging two extraocular muscles or connecting an extraocular muscle to the orbit. Wang et al. [6] reported that accessory extraocular muscles are commonly observed connecting the IR to the posterior sclera, as well as bridging the superior rectus muscle and MR. In this case, the accessory extraocular muscle was located between the IR and MR, bridging the MR and posterior sclera. Compared to previous reports, this represents a rare anatomical variant.

CCDDs often present with abnormal MRI findings in both the cranial nerves and extraocular muscles, including changes in the thickness of the oculomotor and abducens nerves and atypical morphology of the extraocular muscles [7]. In the present case, the patient showed atypical strabismus characterized by limited abduction and supraduction, as well as downshoot during attempted abduction. Based on these clinical features, Duane’s syndrome was initially suspected; therefore, imaging of the orbit was obtained before strabismus surgery to identify potential causes of the condition and develop an appropriate surgical plan.

In this case, although the accessory extraocular muscle was close to the optic nerve, it did not involve the nerve directly. Because the optic nerve shifts temporally during adduction [8], the muscle remained relatively distant from the nerve during the surgical approach, and the risk of optic nerve injury was considered lower than in previous reports [4,5]. The nasal location of the muscle also allowed direct dissection in adduction, ensuring a safe procedure. Although the surgery was successful in this case, we cannot assume that surgical outcomes would be equally favorable in cases where the accessory muscle is situated closer to the optic nerve. Goyal et al. [9] recently reported the use of three-dimensional reconstruction modeling to better understand the anatomical relationships of accessory extraocular muscles. Collaboration between anatomical and engineering disciplines may further enhance the safety and precision of surgical approaches in such complex cases.

## Conclusions

We encountered a case of ET caused by an accessory extraocular muscle, diagnosed on MRI. Subsequent postoperative MRI confirmed the release of traction by the accessory extraocular muscle and improvement in ocular alignment. Because strabismus surgery involving an accessory extraocular muscle carries the added risk of visual impairment due to optic nerve damage, it is crucial to perform a comprehensive preoperative assessment and exercise caution in selecting appropriate patients.

## References

[1] Whitnall SE (1911). An instance of the retractor bulbi muscle in man. J Anat Physiol.

[2] Lueder GT (2002). Anomalous orbital structures resulting in unusual strabismus. Surv Ophthalmol.

[3] Khitri MR, Demer JL (2010). Magnetic resonance imaging of tissues compatible with supernumerary extraocular muscles. Am J Ophthalmol.

[4] Molinari A, Plager D, Merino P, Galan MM, Swaminathan M, Ramasuramanian S, de Faber JT (2016). Accessory extraocular muscle as a cause of restrictive strabismus. Strabismus.

[5] Wang X, Shen T, Han M, Yan J (2022). Supernumerary extraocular muscle: a rare cause of atypical restrictive strabismus. Medicina (Kaunas).

[6] Wang D, Yang YY, Chang QL, Ma Q, Wang YD, Man FY, Jiao YH (2025). Congenital restrictive strabismus associated with anomalous orbital structures: MRI findings and clinical characteristics. Am J Ophthalmol.

[7] Demer JL, Ortube MC, Engle EC, Thacker N (2006). High-resolution magnetic resonance imaging demonstrates abnormalities of motor nerves and extraocular muscles in patients with neuropathic strabismus. J AAPOS.

[8] Smiddy WE, Michels RG, Kumar AJ (1989). Magnetic resonance imaging of retrobulbar changes in optic nerve position with eye movement. Am J Ophthalmol.

[9] Goyal P, Tibrewal S, Lefebvre DR, Ganesh S, Hunter DG (2024). Challenges in management of congenital enophthalmos due to anomalous accessory orbital extraocular muscle bands. Strabismus.

